# Fuzheng Huayu Recipe Ameliorates Liver Fibrosis by Restoring Balance between Epithelial-to-Mesenchymal Transition and Mesenchymal-to-Epithelial Transition in Hepatic Stellate Cells

**DOI:** 10.1155/2015/935903

**Published:** 2015-12-31

**Authors:** Qin Pan, Yu-Qin Wang, Guang-Ming Li, Xiao-Yan Duan, Jian-Gao Fan

**Affiliations:** ^1^Department of Gastroenterology, Xinhua Hospital, Shanghai Jiaotong University School of Medicine, Shanghai 200092, China; ^2^Shanghai Key Laboratory of Children's Digestion and Nutrition, Shanghai 200092, China

## Abstract

Activation of hepatic stellate cells (HSCs) depending on epithelial-to-mesenchymal transition (EMT) reflects the key event of liver fibrosis. Contrastively, mesenchymal-to-epithelial transition (MET) of HSCs facilitates the fibrosis resolution. Here we investigated the effect of Fuzheng Huayu (FZHY) recipe, a Chinese herbal decoction made of* Radix Salviae Miltiorrhizae*,* Semen Persicae*,* Cordyceps sinensis*,* Pollen Pini*, and* Gynostemma pentaphyllum*, on liver fibrosis concerning the balance of EMT and MET in HSCs. In contrast to the increased TGF-*β*_1_/BMP-7 ratio in activated HSCs, FZHY administration induced significant upregulation of BMP-7 and downregulation of TGF-*β*_1_ at both transcription and translation levels. Restoration of TGF-*β*_1_/BMP-7 ratio inhibited the expression of p38 MAPK and phosphorylated p38 MAPK, resulting in the reversal of epithelial-to-mesenchymal transition (EMT) to mesenchymal-to-epithelial transition (MET) as characterized by the abolishment of EMT markers (*α*-SMA and desmin) and reoccurrence of MET marker (E-cadherin).* In vivo* treatment of FZHY recipe also demonstrated the statistical reduction of activated HSCs with EMT phenotype, which attenuated the carbon tetrachloride- (CCl_4_-) induced liver fibrosis in a dose-dependent manner. These findings may highlight a novel antifibrotic role of FZHY recipe on the basis of rebalancing EMT and MET in HSCs.

## 1. Introduction

Hepatic fibrosis represents the common outcome of chronic liver diseases, such as chronic viral infections, alcoholism, fatty liver diseases, and cholestasis, and associates with both hepatocellular carcinoma (HCC) and hepatic failure. Activation of hepatic stellate cells (HSCs) has been well established to contribute to most fibrosis-related characteristics [1].

Nowadays, Fuzheng Huayu (FZHY) recipe, a decoction of* Radix Salviae Miltiorrhizae*,* Semen Persicae*,* Cordyceps sinensis*,* Pollen Pini*, and* Gynostemma pentaphyllum*, is reported to exert antifibrosis actions in several clinical trials [2–5]. Three-month treatment of FZHY recipe gives rise to the decreased serum levels of procollagen peptides and laminin in 63 patients with chronic hepatitis B (CHB). Liver biopsy uncovers the remarkable amelioration of fibrosis grade in most of them [2]. Similar results are detected in another multicentral clinical trial for 180 patients with CHB. Significant improvements in serum level of collagen type IV and fibrosis scoring confirm the therapeutic role of FZHY recipe in hepatic fibrosis [3]. Although FZHY recipe exhibits the prospect for fibrosis prevention, mechanisms underlying its actions still remain to be clarified.

Recent studies uncover that TGF-*β*_1_, the key mediators of liver fibrosis, induces typical characteristics of epithelial-to-mesenchymal transition (EMT), also known as activation, in HSCs [6, 7]. Accompanied by the loss of epithelial markers and gain of mesenchymal markers, HSCs undergoing EMT demonstrate collagen overproduction and formation of fibrous tissue, which further initiates and perpetuates the liver fibrosis [6]. In contrast, reverse process of EMT, namely, mesenchymal-to-epithelial transition (MET), has recently been proved to counteract TGF-*β*_1_-induced fibrotic response in different organs [8, 9]. BMP-7, one of the members of TGF-*β* surperfamily, acts as the critical mediator of MET [10]. Therefore, balance between EMT and MET of HSCs is suggested to determine the liver fibrosis on a basis of, at least to a large extent, TGF-*β*_1_/BMP-7 ratio. Surprisingly, there are growing evidences that FZHY recipe exerts intensive impact on the TGF-*β*_1_/BMP-7 ratio, and also the downstream signaling. The FZHY recipe has already been identified to downregulate the hepatic expression of TGF-*β*_1_ [11] and strongly inhibits the EMT-specific signal in renal interstitial cells [4]. Thus FZHY recipe is hypothesized to effect a pattern of EMT/MET rebalancing by modulating the TGF-*β*_1_/BMP-7 ratio.

To verify the role of FZHY recipe in the balance between EMT and MET, primary rat HSCs were activated and incubated with FZHY-containing serum. The expression of both TGF-*β*_1_ and BMP-7 in HSCs was evaluated according to their transcription and translation levels. Analysis of p38 MAPK, with and without phosphorylation, reflected the downstream signaling depending on TGF-*β*_1_/BMP-7 ratio. Markers specific to epithelial and mesenchymal cells further characterized the status of EMT and MET. Finally, experimental liver fibrosis was subjected to FZHY treatment so as to reveal the consequence of EMT/MET rebalancing.

## 2. Materials and Methods

### 2.1. Cell Preparation

**Figure 1:**
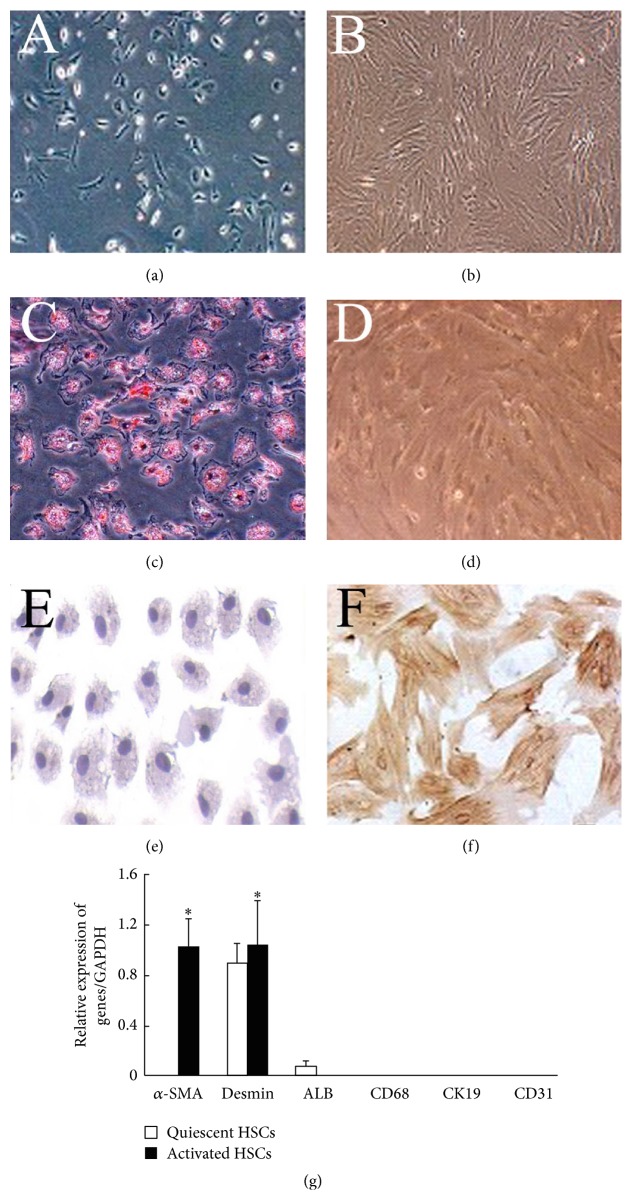
Characterization of HSCs isolated from rat liver. (a, b) Cell morphology of quiescent (a) and activated HSCs (b) (400x). (c, d) Oil Red O staining of quiescent (c) and activated HSCs (d) (400x). (e, f) Immunocytochemistry staining of quiescent (e) and activated HSCs for *α*-SMA (f) (400x). (g) Real-time PCR for ALB, *α*-SMA, CD31, CD68, CK-19, and desmin, respectively, demonstrated the purity of quiescent and activated HSCs. ^*∗*^ *P* < 0.05 compared with quiescent HSCs.

HSCs were separated from adult male Sprague-Dawley (SD) rats (400–500 g, Experimental Animal Center of Chinese Academy of Science, Shanghai, China) by* in situ* serial infusion with D-Hanks' and perfusion medium (Hanks' medium containing 0.05% collagenase IV and 0.1% Pronase E), redigestion with collagenase IV and DNase, and single-step density gradient centrifugation with 18% Nycodenz (W/V) [4]. Their living percentage was up to 95% as defined by staining of trypan blue, while their purity was over 90% when assessed by Oil Red O staining and real-time PCR for *α*-smooth muscle actin (*α*-SMA), desmin, albumin (ALB), CK19, CD31, and CD68, respectively [1] (Figures 1(a)–1(d), 1(g)). Immunocytochemical staining of *α*-SMA (Santa Cruz, USA) denoted the spontaneous activation of nearly all HSCs on the 10th day (Figures 1(e) and 1(f)).

The rats received humane care in accordance with the Declaration of Helsinki (1964). The study was approved by the ethical committee of Shanghai Jiaotong University School of Medicine.

### 2.2. FZHY Administration

Adult male SD rats were treated by FZHY recipe (Pearl Ocean Pharmaceutical Holdings Limited, Beijing, China) intragastrically (10-fold of the maximum daily human dosage of 65 kg, every 2 hours for 3 times). Blood was collected from inferior vena cava 2 hours after the last administration. FZHY-containing serum was then obtained by centrifugation (3000 g for 10 minutes) and inactivation (56°C for 30 min) [12].

Activated HSCs in logarithmic growth phase were randomized into groups of model, 5% FZHY treatment, 10% FZHY treatment, and 20% FZHY treatment. Quiescent HSCs were used as the control group. All FZHY-treated groups were incubated with FZHY-containing serum for 48 h, while saline at the same volume was added into the control and model groups.

### 2.3. Real-Time PCR

Total RNA, being extracted from HSCs, was subjected to RT reaction by ExScript RT reagent kit (TAKARA, Kusatsu, Japan). Real-time PCR was then performed using SYBR Premix Ex Taq (TAKARA, Kusatsu, Japan) on a Light Cycler (Roche Diagnostics GmbH, Penzberg, Germany) according to the manufacturer's instructions. Primer sequences and endogenous control for these reactions were exhibited in Table 1. Relative gene expression levels were calculated by the 2^−ΔΔCT^ method [13].

### 2.4. Western Blot

Total protein of each sample was prepared by standard procedures and quantified by the bicinchoninic acid method (Pierce, Rockford, USA). These protein samples were loaded on 10% polyacrylamide gels and electrophoretically separated. Subsequently, the protein was transferred to polyvinilidene difluoride membranes (Millipore, USA), blocked with 0.1% Tween 20 (TBST) in 5% nonfat dry milk (NFDM) based on Tris-buffered saline (TBS), reacted with primary antibodies (anti-TGF *β*_1_ (1 : 500), anti-BMP-7 (1 : 1000), anti-p38 MAPK (1 : 1500), anti-phosphorylated-p38 MAPK (anti-p-p38 MAPK, 1 : 1000), anti-E-cadherin (1 : 500), anti-*α*-SMA (1 : 1000), and anti-desmin (1 : 600), Santa Cruz, USA) overnight at 4°C and then HRP-conjugated goat anti-mouse IgG (1 : 3000; Jackson ImmunoResearch Laboratories, Inc., USA) for 2 h at room temperature. After washing, the membrane was processed using Super-Signal West Pico Chemiluminescent Substrate (Pierce), and antibody against GAPDH (Santa Cruz, USA) (1 : 1000) as an internal control [14].

### 2.5. Animals and Grouping

Adult male SD rats were randomly divided into normal control (*n* = 10), model (*n* = 10), and FZHY-treated group (*n* = 10). Except for those in the normal control group, all rats were subcutaneously injected with 40% CCl_4_ (0.3 mL/100 g) every 3 days for 6 weeks. Rats of the normal control group received same volume of olive oil in the same way [15]. During the exposure to CCl_4_, FZHY recipe dilution (75 mg/mL of FZHY recipe) was intragastrically administered to the low-dose (2.5 mL/kg/d), medium-dose (5 mL/kg/d), and high-dose (10 mL/kg/d) FZHY-treated rats for 4 weeks, while saline was administered to rats in the model group [16].

### 2.6. Immunohistochemistry

Liver specimens of all groups were prepared and subjected to the following procedure: (1) inhibition of endogenous peroxidase activity by 3% H_2_O_2_ for 15 minutes; (2) digestion by 0.01% trypsin for 10 minutes; (3) blocking nonspecific binder by bovine albumin for 10 minutes; (4) incubation with anti-*α*-SMA (1 : 100, Santa Cruz, USA) overnight at 4°C, (5) reaction with biotin-conjugated secondary antibody (1 : 200; Jackson ImmunoResearch Laboratories, Inc., USA) for 30 minutes; (6) visualization by DAB kit [17].

### 2.7. Hydroxyproline Assay

Liver samples were hydrolyzed in HCl (6 mol/L) at 120°C for 16 h. After being neutralized with NaOH (6 mol/L), the supernatant was oxidized with Chloramine T (Sigma-Aldrich Corp., USA) in acetate/citrate buffer and mixed with Ehrlich's solution. The mixture was then incubated at 60°C for 30 min, and its absorbance was determined at 560 nm [18].

### 2.8. Histology Assay

Histological characteristics, including hepatocyte steatosis, inflammation, and fibrosis, of liver tissue were investigated by both hematoxylin-eosin (H&E) and Van Gieson (VG) staining. Liver fibrosis was evaluated according to Metavir stage [19]. Obtained results were independently interpreted by 2 pathologists, who were not aware of the study.

### 2.9. Statistical Analysis

Data are expressed as means ± standard deviation (SD). All groups were compared statistically by one-way ANOVA using SPSS 16.0 statistical package. Differences with *P* < 0.05 were considered statistically significant.

## 3. Results

### 3.1. FZHY Recipe Recovered the TGF-*β*_1_/BMP-7 Ratio in Activated HSCs

**Figure 2:**
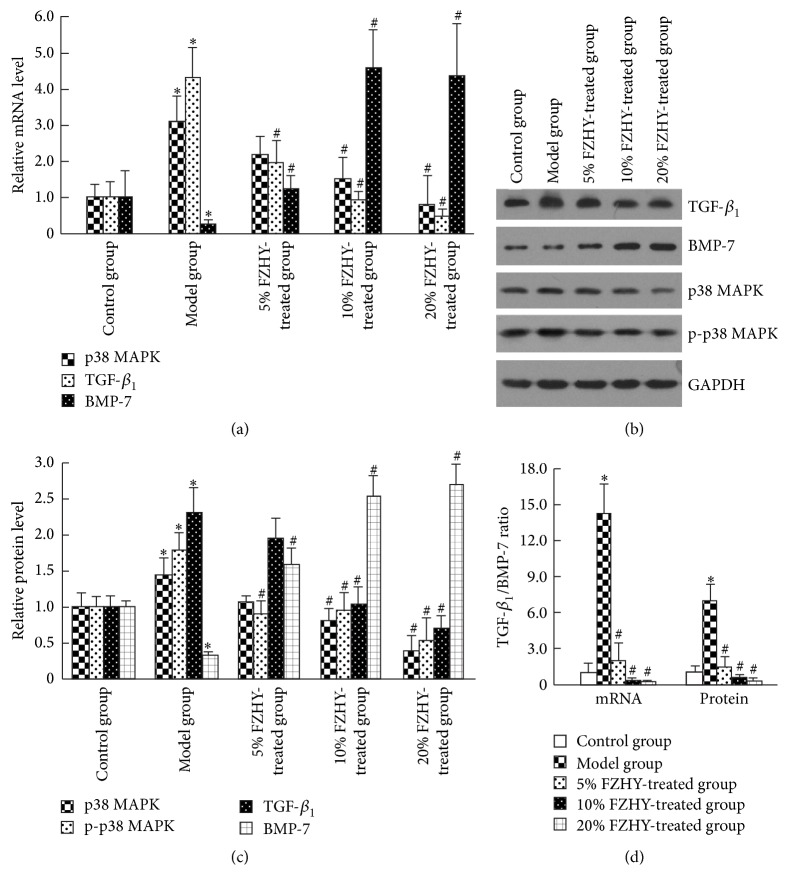
Normalization of TGF-*β*_1_/BMP-7 ratio by FZHY recipe inhibits p38 MAPK signaling pathway in activated HSCs. (a) The mRNA expression levels of TGF-*β*_1_, BMP-7, and p38 MAPK in the control, model, 5% FZHY-treated, 10% FZHY-treated, and 20% FZHY-treated group, respectively. (b) Western blot of TGF*β*_1_, BMP-7, p38 MAPK, and p-p38 MAPK in the control, model, 5% FZHY-treated, 10% FZHY-treated, and 20% FZHY-treated group, respectively. (c) Relative protein levels of TGF*β*_1_, BMP-7, p38 MAPK, and p-p38 MAPK. (d) The TGF-*β*_1_/BMP-7 ratio at transcriptional and translational levels. ^*∗*^ *P* < 0.05 compared with control group. ^#^ *P* < 0.05 compared with model group.

When compared to those of quiescent HSCs (control group), significant increase in the mRNA and protein levels of TGF-*β*_1_ was detected in activated HSCs (model group) (*P* < 0.05, Figures 2(a)–2(c)). Meanwhile, the mRNA and protein levels of BMP-7 were dramatically downregulated in activated HSCs (control group versus model group, *P* < 0.05). In contrast, treatment of FZHY-containing serum dose-dependently reduced the mRNA level of TGF-*β*_1_, whereas it restored the mRNA level of BMP-7 (Figure 2(a)). Western blot further confirmed that the changes in TGF-*β*_1_ and BMP-7 transcription were paralleled by 3.29-fold decrease in TGF-*β*_1_ protein expression (model group versus 20% FZHY-treated group, *P* < 0.05) and 7.71-fold increase in BMP-7 protein expression (model group versus 20% FZHY-treated group, *P* < 0.05) after the treatment of FZHY recipe (Figures 2(b) and 2(c)).

According to the expression of TGF-*β*_1_ and BMP-7, there was statistical upregulation of TGF-*β*_1_/BMP-7 ratio in activated HSCs at both transcriptional and translational levels (control group versus model group, *P* < 0.05, Figure 2(d)). However, administration of FZHY recipe, no matter the 5% FZHY-, 10% FZHY-, and 20% FZHY-treated groups, resulted in the normalization of TGF-*β*_1_/BMP-7 ratio (Figure 2(d)), suggesting a regulatory role in the balance of EMT and MET.

### 3.2. Restoration of TGF-*β*_1_/BMP-7 Ratio Attenuated EMT and Induced MET of HSCs via p38 MAPK Signaling

**Figure 3:**
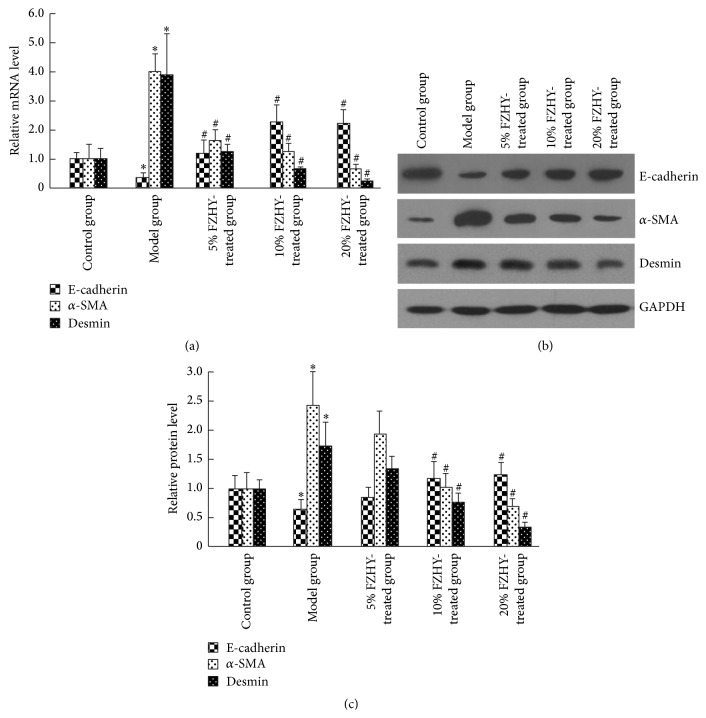
Inactivation of p38 MAPK signaling pathway prevents EMT and induced MET of HSCs. (a) The mRNA expression levels of E-cadherin (MET specific gene), *α*-SMA (EMT specific gene), and desmin (EMT specific gene) in the control, model, 5% FZHY-treated, 10% FZHY-treated, and 20% FZHY-treated group, respectively. (b) Western blot of E-cadherin, *α*-SMA, and desmin in the control, model, 5% FZHY-treated, 10% FZHY-treated, and 20% FZHY-treated group, respectively. (c) Relative protein levels of E-cadherin, *α*-SMA, and desmin. ^*∗*^ *P* < 0.05 compared with control group. ^#^ *P* < 0.05 compared with model group.

To assess the effect of TGF-*β*_1_/BMP-7 ratio on EMT/MET balance, we evaluated the downstream signaling of p38 MAPK, and then epithelial marker (E-cadherin) and mesenchymal markers (*α*-SMA and desmin) in quiescent, activated, and FZHY-treated HSCs, respectively. In contrast to the greatly upregulated transcription of p38 MAPK, *α*-SMA, and desmin in activated HSCs (control group versus model group, *P* < 0.05), their mRNA levels reduced after FZHY administration in a dose-dependent manner (Figures 2(a), 3(a)). On the contrary, the E-cadherin expression of activated HSCs was over 64.51% lower than the normal level (control group versus model group, *P* < 0.05), while treatment of FZHY recipe led to about 6.48-fold upregulation in its mRNA level (model group versus 20% FZHY-treated group, *P* < 0.05) (Figure 3(a)). Similarly to the changes in transcription, declined protein level of E-cadherin and increased protein levels of p38 MAPK, p-p38 MAPK, *α*-SMA, and desmin characterized the activation of HSCs (control group versus model group, *P* < 0.05) (Figures 2(b) and 2(c), 3(b) and 3(c)). However, FZHY-treated HSCs, with exposure to 10% and 20% FZHY-containing serum for 48 hours, demonstrated p38 MAPK, p-p38 MAPK, E-cadherin, *α*-SMA, and desmin levels comparable with those of quiescent HSCs (Figures 2(b) and 2(c), 3(b) and 3(c)). Thus the p38-dependent recovery from EMT to MET was indicated in FZHY-treated HSCs.

### 3.3. Rebalance of EMT and MET by FZHY Administration Abolished HSCs with Activated Phenotype* In Vivo*

**Figure 4:**
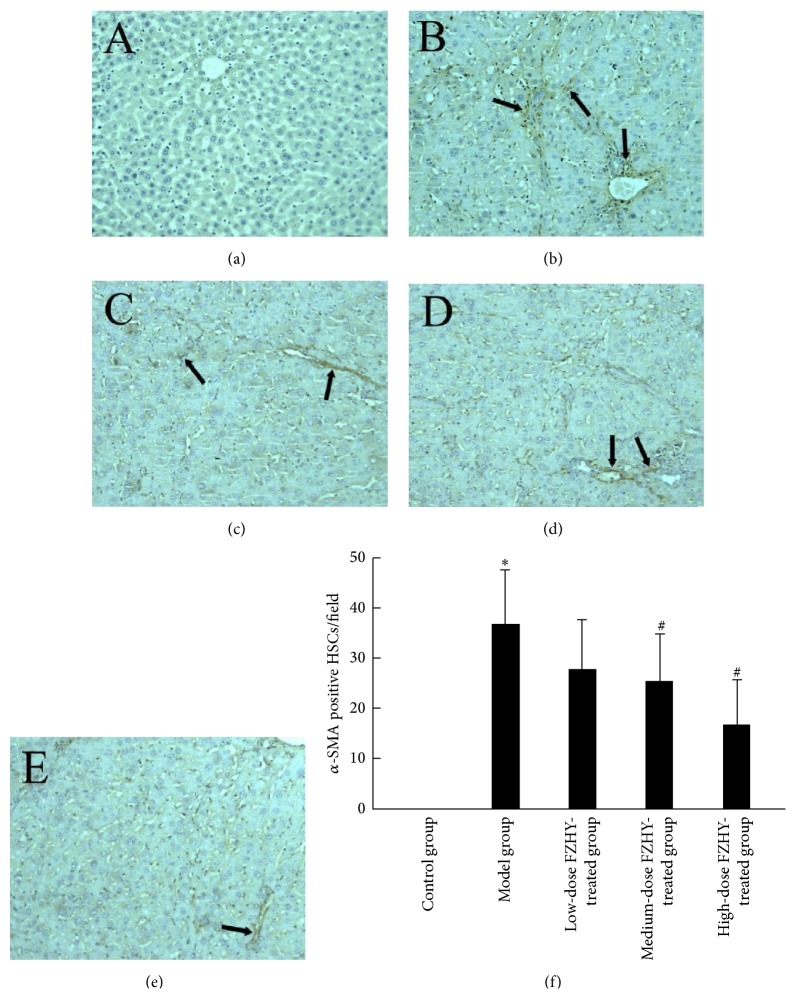
FZHY recipe abolishes the EMT-dependent activation of HSCs. Arrows indicate the immunohistochemical staining of *α*-SMA in the control group (a), model group (b), low-dose FZHY-treated group (c), medium-dose FZHY-treated group (d), and high-dose FZHY-treated group (e), respectively (×200). (f) Quantification of *α*-SMA-positive HSCs in different groups. ^*∗*^ *P* < 0.05 compared with control group. ^#^ *P* < 0.05 compared with model group.

No *α*-SMA positive cell was found in hepatic lobules of normal control group, indicating no activated HSC existed. However, numerous *α*-SMA positive cells with spindle or asteroid shape, most of which located in sinusoidal space, central vein, and fibrous tissue, were observed in rats exposure to CCl_4_ for 6 weeks (model group, Figure 4). At higher magnification of microscopy, the number of *α*-SMA positive cells rised up to 36.7 ± 11.4 per field (control group versus model group, *P* < 0.05). Dramatically, only 16.9 ± 8.9  *α*-SMA positive cells per field could be detected in the high-dose FZHY-treated group, which was significantly lower than that in the model group (*P* < 0.05, Figure 4).

### 3.4. Treatment of FZHY Recipe Inhibited CCl_4_-Induced Liver Fibrosis

**Figure 5:**
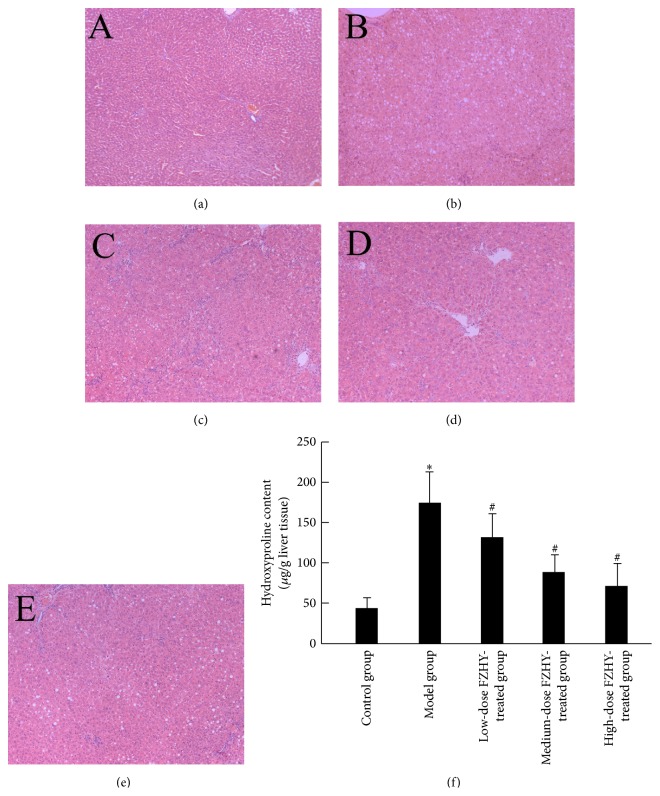
Administration of FZHY recipe ameliorates the CCl_4_-induced pathological disorders in liver. (a–e) H&E staining of the control group (a), model group (b), low-dose FZHY-treated group (c), medium-dose FZHY-treated group (d), and high-dose FZHY-treated group (e), respectively (×100). (f) Hepatic hydroxyproline content of different groups. ^*∗*^ *P* < 0.05 compared with control group. ^#^ *P* < 0.05 compared with model group.

When compared with the normal control group, hepatic micro- and macrovesicular steatosis characterized the model group after CCl_4_ exposure. Other pathological disorders, including ballooning degeneration of hepatocytes, infiltration of inflammatory cells in lobules, and active proliferation of myofibroblast, were also visualized (Figure 5). Contrastively, most of these alternations were attenuated after different dosage of FZHY treatment (Figure 5).

**Figure 6:**
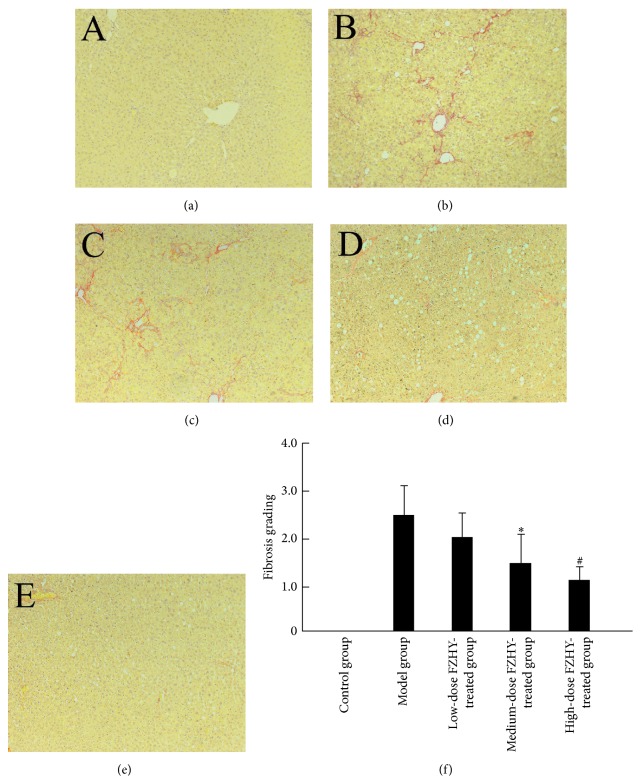
FZHY treatment attenuates CCl_4_-induced liver fibrosis. (a–e) VG staining of the control group (a), model group (b), low-dose FZHY-treated group (c), medium-dose FZHY-treated group (d), and high-dose FZHY-treated group (e), respectively (×100). (f) Metavir score of liver fibrosis in different groups. ^*∗*^ *P* < 0.05 compared with model group. ^#^ *P* < 0.01 compared with model group.

The VG staining uncovered similar phenomenon that little fibrosis existed in the liver of normal control rats, while excessive collagen deposition and well-delineated fibrosis septa emerged in the model group. Fibrous tissue is widely distributed in sinusoidal space and lobule, sometimes even bridging central vein and portal regions (Figure 6). After the treatment of FZHY recipe for 4 weeks, Metavir scores revealed the great improvement of liver fibrosis from 2.49 ± 0.61 (model group) to 1.57 ± 0.49 (medium-dose FZHY-treated group, *P* < 0.05), and 1.08 ± 0.33 (high-dose FZHY-treated group, *P* < 0.01) (Figure 6).

## 4. Discussion

Recently, increased studies have revealed the potential of FZHY recipe in prevention and treatment of organic fibrosis, such as hepatic, renal interstitial, and myocardial fibrosis [2–5, 11]. Similar to other traditional Chinese prescriptions, a variety of mechanisms are now recognized to underlie the effect of FZHY recipe, which includes (1) preventing HSCs from activation, as well as promoting their apoptosis; (2) inhibiting the proliferation of HSCs; (3) downregulating the expression and secretion of collagen; (4) inducing the activation of matrix metalloproteases; (5) protecting hepatocytes from apoptosis and facilitating their proliferation; (6) improving the liver function test; and (7) normalizing the proteome of liver tissue [2, 3, 5, 11]. However, these complicated mechanisms make it a difficult task to identify the comprehensive effect of FZHY recipe.

Balance of EMT and MET takes a central place in the activation of HSCs. HSCs which underwent EMT demonstrate most fibrosis-inducing characteristics, including migration towards the lesions, secretion of cytokines (TGF-*β*_1_, PDGF, etc.), and synthesis of ECM (collagen, laminine, hyaluronic acid, etc.) [20, 21]. MET represents the counteracting process against EMT-related fibrotic response [9]. Nowadays, transdifferentiation between EMT and MET is indicated to be tightly controlled by cytokines, especially TGF-*β*_1_ and BMP-7 [6]. After being incubated with FZHY-containing serum for 48 hours, HSCs showed dose-dependent reduction of TGF-*β*_1_ expression in the 10% and 20% FZHY-treated groups. On the other hand, both the mRNA and protein levels of BMP-7, which experienced expression loss during EMT, upregulated significantly in the HSCs of 5%, 10%, and 20% FZHY-treated groups. TGF-*β*_1_/BMP-7 ratio was finally normalized in the groups of FZHY administration, mimicking that of the control group. Therefore, FZHY recipe may exert its effects via regulating TGF-*β*_1_/BMP-7 ratio in a dose-dependent manner.

MAPK signaling acts as the common pathway downstream to TGF-*β*_1_ and BMP-7 stimulation [22]. p38 MAPK among MAPK family has been proved to mediate the TGF-*β*_1_-induced synthesis of procollagen I in murine glomerular mesangial cells and seems to be indispensible for the glomerular sclerosis [23, 24]. Moreover, activation of p38 MAPK plays an essential role in the TGF-*β*_1_-induced expression of mesenchymal marker (*α*-SMA) in human vascular smooth muscle cells [25]. Thus activation of p38 MAPK signaling, partially in the form of phosphorylated p38 MAPK, may reflect the key step from high TGF-*β*_1_/BMP-7 ratio to EMT/MET unbalance. In contrast to their limited expression in the control group, elevated p38 MAPK and p-p38 MAPK in the model group indeed characterized the activation of p38 MAPK signaling during EMT of HSCs in our experiments. Dramatically, treatment of 10% and 20% FZHY-containing serum effectively inactivated p38 MAPK signaling by downregulating the expression and phosphorylation of p38 MAPK.

To shed light on the p38-mediated effect on phenotypic regulation, markers specific to mesenchymal and epithelial cells were subjected to investigation. E-cadherin, an epithelial cell specific transmembrane glycoprotein, forms complex with *α*-catenin, *β*-catenin, and catenin p120, which maintains the polarity of quiescent HSCs by binding to skeleton proteins. Significant decrease of E-cadherin contributes to the cytoskeleton rearrangement and polarity loss, and then EMT of HSCs [26]. In the present study, both the mRNA and protein levels of E-cadherin were significantly higher in the 10% and 20% FZHY-treated groups rather than those in the model group. Opposite to its promotional action on E-cadherin, treatment of FZHY-containing serum led to the inhibition of mesenchymal markers in HSC [27]. When determined with QPCR and Western blot, the expression levels of *α*-SMA and desmin were significantly lowered in the 5%, 10%, and 20% FZHY-treated groups than the controls. Moreover, the level of *α*-SMA and desmin proteins in the 10% and 20% FZHY-treated groups were in parallel to the normal control group, while the mRNA of *α*-SMA and desmin in these two groups were even lower than those in the normal control group. These observations qualify FZHY for a potential recipe to abrogate EMT and induce MET in HSCs.

Facilitating the transdifferentiation from EMT to MET, FZHY recipe is likely to ameliorate liver fibrosis by means of removing HSCs with EMT characteristics.* In vivo* treatment of FZHY recipe was then performed in the rodent model of CCL_4_-induced liver fibrosis. When compared to that of the control group, there were much more *α*-SMA-positive HSCs in the fibrous septum and sinusoid of the model group. FZHY therapy, however, statistically reduced these EMT signals specific to fibrosis-inducing HSCs. Being consistent with previous reports [2, 3, 11], attenuation of liver fibrosis was resultantly achieved by FZHY recipe. The Metavir score in high-dose FZHY-treated group was only 1.08 ± 0.33, which was significantly lower than that in the model group (2.49 ± 0.61, *P* < 0.05).

## 5. Conclusions

FZHY recipe may downregulate the TGF-*β*_1_/BMP-7 ratio and, subsequently, result in the reversal of EMT to MET in activated HSCs via the inhibition of p38 MAPK signaling pathway. The rebalance of EMT and MET by FZHY recipe serves as a novel resolution of liver fibrosis on the basis of reducing HSCs with EMT characteristics.

## Figures and Tables

**Table 1:** Primers for real-time PCR.

Gene	Primer sequence (5′-3′)	Annealing temperature (°C)	Product length (bp)
ALB	Forward primer: GACTGCCCTGTGTGGAAGAC Reverse primer: CGAAGTCACCCATCACCGTC	56	321

*α*-SMA	Forward primer: GCGGGGATCCATGAGACCACCTAC Reverse primer: GAGCCGCCGATCCACACTGA	60	218

CK19	Forward primer: CACTACGCAGATCCAGATAAACA Reverse primer: GAAGTCGCACTGGTAGCAAG	56	388

CD31	Forward primer: CTTCACCATCCAGAAGGAAGAGAC Reverse primer: CACTGGTATTCCATGTCTCTGGTG	56	360

CD68	Forward primer: TCATGGGAATGCCACAGTTTC Reverse primer: GAGGGCCAACAGTGGAGAA	56	62

Desmin	Forward primer: GGCCGACGCAGTGAACCAG Reverse primer: CGCCGCAGCTCTCGCATCT	60	213

E-cadherin	Forward primer: TGAAGCCCCCATTTTTGTGC Reverse primer: GCTCCGAATCTTCTCTGTCCATCT	58	221

TGF-*β*_1_	Forward primer: GCGCCTGCAGAGATTCAAGTCA Reverse primer: AAAGCCCTGTATTCCGTCTCCT	56	187

GAPDH	Forward primer: CACGGCAAGTTCAACGGCACAGT Reverse primer: AGCGGAAGGGGCGGAGATGAT	60	222

## References

[1] Guo C.-J., Pan Q., Li D.-G., Sun H., Liu B.-W. (2009). miR-15b and miR-16 are implicated in activation of the rat hepatic stellate cell: an essential role for apoptosis. *Journal of Hepatology*.

[2] Song Y.-N., Sun J.-J., Lu Y.-Y. (2013). Therapeutic efficacy of fuzheng-huayu tablet based traditional Chinese medicine syndrome differentiation on hepatitis-B-caused cirrhosis: a multicenter double-blind randomized controlled trail. *Evidence-Based Complementary and Alternative Medicine*.

[3] Liu P., Hu Y.-Y., Liu C. (2005). Multicenter clinical study on Fuzhenghuayu capsule against liver fibrosis due to chronic hepatitis B. *World Journal of Gastroenterology*.

[4] Wang Q.-L., Yuan J.-L., Tao Y.-Y., Zhang Y., Liu P., Liu C.-H. (2010). Fuzheng Huayu recipe and vitamin E reverse renal interstitial fibrosis through counteracting TGF-*β*1-induced epithelial-to-mesenchymal transition. *Journal of Ethnopharmacology*.

[5] Liu C., Liu P., Liu C.-H., Zhu X.-Q., Ji G. (1998). Effects of Fuzhenghuayu decoction on collagen synthesis of cultured hepatic stellate cells, hepatocytes and fibroblasts in rats. *World Journal of Gastroenterology*.

[6] Firrincieli D., Boissan M., Chignard N. (2010). Epithelial-mesenchymal transition in the liver. *Gastroenterologie Clinique et Biologique*.

[7] Bi W.-R., Yang C.-Q., Shi Q. (2012). Transforming growth factor-*β*1 induced epithelial-mesenchymal transition in hepatic fibrosis. *Hepato-Gastroenterology*.

[8] Vargha R., Endemann M., Kratochwill K. (2006). Ex vivo reversal of in vivo transdifferentiation in mesothelial cells grown from peritoneal dialysate effluents. *Nephrology Dialysis Transplantation*.

[9] Xie G., Diehl A. M. (2013). Evidence for and against epithelial-to-mesenchymal transition in the liver. *American Journal of Physiology—Gastrointestinal and Liver Physiology*.

[10] Yang G., Zhu Z., Wang Y., Gao A., Niu P., Tian L. (2013). Bone morphogenetic protein-7 inhibits silica-induced pulmonary fibrosis in rats. *Toxicology Letters*.

[11] Liu P. (2012). Fuzheng huayu capsule in the treatment of liver fibrosis: clinical evidence and mechanism of action. *Chinese Journal of Integrative Medicine*.

[12] Iwama H., Amagaya S., Ogihara Y. (1987). Effect of shosaikoto, a Japanese and Chinese traditional herbal medicinal mixture, on the mitogenic activity of lipopolysaccharide: a new pharmacological testing method. *Journal of Ethnopharmacology*.

[13] Livak K. J., Schmittgen T. D. (2001). Analysis of relative gene expression data using real-time quantitative PCR and the 2^−ΔΔ*C*_T_^ method. *Methods*.

[14] Lee D. C., Stenland C. J., Hartwell R. C. (2000). Monitoring plasma processing steps with a sensitive Western blot assay for the detection of the prion protein. *Journal of Virological Methods*.

[15] Guo C.-J., Pan Q., Cheng T., Jiang B., Chen G.-Y., Li D.-G. (2009). Changes in microRNAs associated with hepatic stellate cell activation status identify signaling pathways. *FEBS Journal*.

[16] Liu Y., Liu P., Hu Y.-Y. (2006). Dynamic change of metabolism related protein in liver tissue of rats' model of hepatic fibrosis and regulatory effect of fuzheng huayu decoction on it. *Zhongguo Zhong Xi Yi Jie He Za Zhi*.

[17] Cassiman D., Denef C., Desmet V. J., Roskams T. (2001). Human and rat hepatic stellate cells express neurotrophins and neurotrophin receptors. *Hepatology*.

[18] Lee H.-S., Shun C.-T., Chiou L.-L., Chen C.-H., Huang G.-T., Sheu J.-C. (2005). Hydroxyproline content of needle biopsies as an objective measure of liver fibrosis: emphasis on sampling variability. *Journal of Gastroenterology and Hepatology*.

[19] Shlomai A., Halfon P., Goldiner I. (2013). Serum bile acid levels as a predictor for the severity of liver fibrosis in patients with chronic hepatitis C. *Journal of Viral Hepatitis*.

[20] Cho I. J., Kim Y. W., Han C. Y. (2010). E-cadherin antagonizes transforming growth factor *β*1 gene induction in hepatic stellate cells by inhibiting RhoA-dependent Smad3 phosphorylation. *Hepatology*.

[21] Chen Y., Luo Q., Xiong Z., Liang W., Chen L., Xiong Z. (2012). Telmisartan counteracts TGF-*β*1 induced epithelial-to-mesenchymal transition via PPAR-*γ* in human proximal tubule epithelial cells. *International Journal of Clinical and Experimental Pathology*.

[22] Yoshikawa M., Hishikawa K., Marumo T., Fujita T. (2007). Inhibition of histone deacetylase activity suppresses epithelial-to-mesenchymal transition induced by TGF-*β*1 in human renal epithelial cells. *Journal of the American Society of Nephrology*.

[23] Chin B. Y., Mohsenin A., Li S. X., Choi A. M. K., Choi M. E. (2001). Stimulation of pro-*α*(1)(I) collagen by TGF-*β*1 in mesangial cells: role of the p38 MAPK pathway. *The American Journal of Physiology—Renal Physiology*.

[24] Yang J., Liu Y. (2001). Dissection of key events in tubular epithelial to myofibroblast transition and its implications in renal interstitial fibrosis. *American Journal of Pathology*.

[25] Martin-Garrido A., Brown D. I., Lyle A. N. (2011). NADPH oxidase 4 mediates TGF-*β*-induced smooth muscle *α*-actin via p38MAPK and serum response factor. *Free Radical Biology and Medicine*.

[26] Izumiya M., Kabashima A., Higuchi H. (2012). Chemoresistance is associated with cancer stem cell-like properties and epithelial-to-mesenchymal transition in pancreatic cancer cells. *Anticancer Research*.

[27] Österreicher C. H., Penz-Österreicher M., Grivennikov S. I. (2011). Fibroblast-specific protein 1 identifies an inflammatory subpopulation of macrophages in the liver. *Proceedings of the National Academy of Sciences of the United States of America*.

